# Defining the “Problem Resident” and the Implications of the Unfixable Problem: The Rationale for a “Front-door” Solution

**DOI:** 10.5811/westjem.2018.11.39867

**Published:** 2018-12-12

**Authors:** Taku Taira, Sally A. Santen, Nicole K. Roberts

**Affiliations:** * LAC+USC Medical Center, Department of Emergency Medicine, Los Angeles, California; † Stony Brook University Medical Center, Department of Emergency Medicine, Stony Brook, New York; ‡ Virginia Commonwealth University School of Medicine, Department of Emergency Medicine, Richmond, Virginia; § The City University of New York (CUNY) School of Medicine, Department of Medical Education, New York, New York

## Abstract

**Introduction:**

Problem residents are common in graduate medical education, yet little is known about their characteristics, deficits, and the consequences for emergency medicine (EM) residencies. The American Board of Internal Medicine (ABIM) defines a problem resident as “a trainee who demonstrates a significant enough problem that requires intervention by someone of authority, usually the program director [PD] or chief resident.” Although this is a comprehensive definition, it lacks specificity. Our study seeks to add granularity and nuance to the definition of “problem resident,” which can be used to guide the recruitment, selection, and training of residents.

**Methods:**

We conducted semi-structured interviews with a convenience sample of EM PDs between 2011 and 2012. We performed qualitative analysis of the resulting transcripts with our thematic analysis based on the principles of grounded theory. We reached thematic sufficiency after 17 interviews. Interviews were coded as a team through consensus.

**Results:**

The analysis identified diversity in the type, severity, fixability, and attribution of problems among problem residents. PDs applied a variety of thresholds to define a problem resident with many directly rejecting the ABIM definition. There was consistency in defining academic problems and some medical problems as “fixable.” In contrast, personality problems were consistently defined as “non-fixable.” Despite the diversity of the definition, there was consensus that residents who caused “turbulence” were problem residents.

**Conclusion:**

The ABIM definition of the problem resident captures trainees who many PDs do not consider problem residents. We propose that an alternative definition of the problem resident would be “a resident with a negative sphere of influence beyond their personal struggle.” This combination acknowledges the identified themes of turbulence and the diversity of threshold. Further, the combination of PDs’ unwillingness to terminate trainees and the presence of non-fixable problems implies the need for a “front-door” solution that emphasizes personality issues at the potential expense of academic potential. This “front-door” solution depends on the commitment of all stakeholders including medical schools, the Association of American Medical Colleges, and PDs.

## INTRODUCTION

Graduate training programs have a responsibility to both the trainee and the public to ensure resident physicians develop the knowledge, skills, and attitudes required to practice medicine independently. Although it is expected that individual trainees will reach Accreditation Council for Graduate Medical Education (ACGME) Milestones at different stages during their training,^1^ some residents will struggle to maintain progress and will need additional resources to meet the established standards of the training program.^2^ Among these struggling residents are a subset that have been labeled “problem residents.” Problem residents challenge educators in graduate medical education with regard to training, remediation, resident and faculty resources, and patient safety.

The American Board of Internal Medicine (ABIM) defines the problem resident as “a trainee who demonstrates a significant enough problem that requires intervention by someone of authority, usually the program director [PD] or chief resident.”^3^ Although the ABIM definition is comprehensive, it lacks granularity, thus prohibiting nuanced discussion and the development of strategies for specific subsets. “Problem residents” account for 7% of all residents and the vast majority of residencies have problem residents.^3^,^4^ While others have classified resident problems in other specialties,^2^,^4^–^7^ there have been no studies to date in emergency medicine (EM) further characterizing the problem resident. The objective of this study was to develop a taxonomy of “problem residents” to inform recruitment, selection, evaluation, and remediation practices.

## METHODS

We employed a constructivist grounded theory approach to our data collection and analysis. This approach requires that the researchers bring their own backgrounds and assumptions to bear on their analysis.^8^ We would like to provide this contextual information. The lead author (TT) worked in residency administration and has been responsible for resident remediation. Two authors (TT and SS) are emergency physicians who work in academic institutions with residencies. Two authors (NR, and SS) have PhDs in education and are employed in the medical school Dean’s office.

One investigator (TT) performed in-depth, semi-structured interviews with a sample of emergency medicine PDs between 2011 and 2012. We employed a purposeful sampling approach to identify the richest data source.^9^ We chose PDs because they have firsthand knowledge of problem residents. Additionally, PDs know the greatest amount of detail about resident actions, remediation, and resolution of problems. We initially recruited current PDs who attended the 2011 Council of Residency Directors (CORD) Academic Assembly meeting. After the initial round of interviews, we again employed purposeful sampling to include PDs with greater experience and to insure adequate sampling of both dually-accredited and three-year programs.

Interviewees were initially asked to describe a specific resident they trained who they considered to be a “problem resident.” The interviewer followed up with questions aimed at obtaining as much detail as possible surrounding the PD’s recall of his/her experience including the resident’s actions, their response, the program and departmental response, and the PD’s attributions and reflections. At the conclusion of the interviews, the PD was asked to define the term “problem resident.” Interviews were recorded and transcribed verbatim by the interviewer. All identifying information was removed from the transcripts.

**Population Health Research Capsule**

What do we already know about this issue?

*“Problem residents” account for 7% of residents in graduate medical education. However, very little is known about how these residents are defined or their characteristics*.

What was the research question?

Our objective was to add granularity to the discussion of “problem residents.”

What was the major finding of the study?

We found a wide range of thresholds for the definition as well as a variety of problems and causes underlying the label “problem residents.”

How does this improve population health?

*Our finding of “unfixable” problems supports the use of a “front-door” approach to the applicant selection process, emphasizing personality over academic performance*.

Each of the interviews was transcribed and uploaded into Atlas.ti^TM^. We used a grounded theory approach (Glaser and Strauss)^10^,^11^ to explore the PD’s description and definitions of the problem resident. We began analyzing transcripts after the initial five interviews were completed. Subsequent interviews were analyzed on completion. Insights from coding informed future interviews using Glaser’s constant comparative method of qualitative analysis.^12^

All coding was done as a group, either in person or over conference calls. One author would read the transcript aloud to the group followed by discussion and coding. We discussed the stories each PD presented as a whole, and then subsequently discussed each semantic unit to reach consensus. As we coded, we tracked emerging themes. We reviewed and consolidated the ensuing code list to develop overarching themes to describe the data. Disagreements were resolved through consensus. Interviews and coding were continued until we reached thematic sufficiency (i.e., until no new insights or codes emerged from the data). This resulted in 17 interviews. This study was approved by the Stony Brook University Medical Center Institutional Review Board.

## RESULTS

The 17 interviews ranged from 11–42 minutes, with an average of 22 minutes per interview. This sample includes PDs from the beginning of their directorship to PDs with more than 20 years of experience with a mean of 8.9 years of experience. Programs included eleven four-year programs, six three-year programs, and two dually-accredited programs.

### Themes

#### Performance

All PDs described problem residents based on a resident’s problematic behaviors in both the clinical and non-clinical areas. Some descriptions were closely aligned with a deficit in a single ACGME competency, while others crossed multiple competencies. We found a continuum of the severity of the problematic behaviors with some PDs describing minor clinical struggles that resulted in no patient harm, while others provided examples of egregious dereliction of duty:

“…over the next month basically ignored 90 charts that were anywhere from 30 to 90 days old […] just ignored them completely. Didn’t respond to emails from me to do them, didn’t respond to emails from the chair to do them, and just didn’t return phone calls, voicemails or text messages…”

Within the theme of performance, the examples centered in the clinical areas, clustered around clinical performance, professionalism, and inter-personal communication. Clinical performance issues touched on multiple EM milestones including medical knowledge, cognitive reasoning, difficulty with the EM acuity, and difficulty with the EM environment. Non-clinical performance problems centered around difficulty with practice-based learning and improvement (PBLI) and non-clinical professionalism.

### Characterizing Problems

PDs characterized problems along two descriptive axes according to their amenability to remediation (fixable/unfixable) and according to their perception of the source of the problem (inherent/acquired).

#### Fixable Problems

Fixable problems were those that could be addressed to the PD and faculty’s satisfaction. These residents graduated with limited or no concerns. Within the fixable problems, PDs most commonly described academic performance problems, especially with test taking. These were typically addressed with reading programs and easily verifiable with performance on in-training examinations and the EM board exam. PDs also described successful interventions on issues ranging from communication, mental health, medical issues, and drug addiction. A PD discussed getting an addicted resident into treatment:

“My very first meeting with him, I had my department administrator sitting with me. I expressed what my concerns were and he expressed in the very next sentence that he was addicted to narcotics and what he was doing is find medication in the sharps box that had not been fully used and had been wasted […] he went off for 3 months and got inpatient therapy and returned to the residency and continued to crush his rotations and the in-training examination and with the help of the physician monitoring apparatus here in [name of state] went on to become licensed and he got a job in his home state in [name of state] and he continues to do well.”

#### Unfixable Problems

PDs were doubtful or unsure if “unfixable” problems were satisfactorily remediated. If residents with “unfixable” problems were allowed to graduate, PD/faculty had ongoing concerns. In contrast to the “fixable” problems, *“*unfixable” problems tended to be associated with personality traits, lack of professionalism, and lack of insight. PDs also described a variety of other issues ranging from poor fit for EM, lack of sufficient intelligence, and lack of motivation to improve. For example, one PD described a resident with a non-fixable problem:

“We just realized that he was unable […] at the end of every shift people would be pissed off at him. The patients were pissed off at him, the nurses were pissed off at him, he had no sense of what he was doing, he agreed into therapy, he had therapy and despite the therapy we saw no movement whatsoever.”

Although there were residents with communication difficulties in both the fixable and unfixable categories, the unfixable problems were attributed to stable traits that were not amenable to intervention.

#### Inherent Problems

The majority of the PD examples were of inherent problems. Inherent problems included areas such as personality problems, communication patterns, lack of intelligence, lack of innate ability, medical/ psychiatric issues, and being a poor fit for EM.

#### Acquired Problems

Acquired problems comprised a minority of the data. These were problems that arose during the course of residency or were stimulated by some external force. The causes of these problems could not be predicted *a piori*. Examples of acquired deficits included issues stemming from neurological injuries, as well as problems at home. Sometimes the distinction between acquired and inherent problems was unclear, often stemming from situations brought on and/or exacerbated by the environment of the emergency department. One PD described addressing a resident’s attention deficit hyperactivity disorder (ADHD):

… finally we sent him for cognitive testing [which] ultimately demonstrated by this testing which was that he had fairly significant ADHD. What’s interesting about this guy was that he was so intelligent and he worked so hard for all of those years during college and medical school that he was able to overcome his capacity to not attend well by working hard at being smart until we basically outstripped his capacity when he was a third year resident […] we flipped the switch with this guy as soon as we got him started on medication he was functional beyond belief […] he was finally able to reach his full potential.

### Threshold

PDs described a wide variability in their threshold for defining a problem resident. Several PDs’ threshold matched the ABIM definition of a resident requiring action, remediation, or intervention.

“…the problem resident […] is the person who requires much more management than the average resident whether that be in the clinical environment because of their cognitive skills or because their interpersonal skills or their systems and professionalism stuff such as turning in things on time, showing up for conference without whatever else…”

Similarly, another PD defined it:

“I would define it as loosely as the residents that sit in my office and I just think… you are killing me… they are memorable for the wrong reasons.”

However, there were many PDs who directly rejected the ABIM definition. One PD noted, “There is no such thing as a problem resident, only problem programs.” Other PDs demonstrated a higher threshold to define a problem resident. These PDs saw intervening to assist trainees as an expected part of residency training:

“…the resident who doesn’t do well on the in-service exam or is bad with compliance. No, I don’t think they are problems at all, I think that they are residents who are on the evolutionary scale that are going to evolve at some point in time but I do not consider them to be problems.”

The threshold for when a non-problem resident turns into a problem resident was PD and program specific. It depended upon how much intervention the PD was willing or able to engage in, the support of the department, the departmental and PD experience with previous success and failure with problem residents, and the PD’s educational philosophy.

### Turbulence

Despite the range of views, the theme of turbulence provided one of the more definitive thresholds. Residents who created turbulence were universally considered problem residents. Turbulence went beyond the minor, commonplace disruption that many residents cause by failing to perform some of the paperwork tasks associated with documenting education (i.e., logging procedures). Instead, these residents caused disruptions that extended beyond the resident and the residency office to impact the department as a whole.

“I would describe her as a problem resident because initially of the amount of disruption that she caused within the program. A few other people characterized her as being like a toxic person and so the effect that she had on other people was toxic. The effect on the program morale was just horrendous.”

Another PD described them this way:

“When I think about the problem residents, the problem resident […] negatively influences, or negatively impacts, you know, causes, for a lack of a better word, turbulence around them.”

The majority of the examples of turbulent residents centered around issues of personality and inter-personal communication.

“Then it seems to continue to get worse and she moved into her final year where she did have a supervisory role and more interns were reporting, were crying just after bad interactions with her and I decided that this is enough of the personality problem here.”

### Resolution

The final theme addressed the resolution of managing the problem resident. PDs were notably hesitant to terminate residents despite knowing that the problem may not be fixable. This was true even with residents who caused significant turbulence. There were a few examples of PDs who terminated residents due to gross misconduct, such as failure of a drug policy, unprofessional behavior, or lack of clinical competency. For the most part, even problem residents graduated and entered independent practice. In these cases, PDs accepted a “good enough” solution.

“…we extended his residency the proper amount of time to get him his training […] then we got him out of the program and we graduated him, and I wrote in his final letter and spoke to his future employers that I felt that a low acuity environment without critical patients, without a trauma center was the place for him…”

Another PD described their “good enough” solution:

“And we were thinking that we would gear it to some level of competence where she could finish the program but not be eligible to sit for the boards and maybe work as an urgent care physician or something like that…”

Many PDs acknowledged the hesitancy and the difficulty with terminating a resident. One PD described their solution as a “front-door solution” which emphasizes not selecting applicants with unfixable problems:

“I haven’t had a resident with real interpersonal problems yet, which is nice. It may [be] attribute[d] to the fact that we do decent screening on our interview days or we do homework on people making calls, but I haven’t had any people with interpersonal problems.”

Another PD said:

“I haven’t had to train any sociopaths; that’s a selection process that’s incumbent on the program director not the applicant.”

## DISCUSSION

In our data, PDs described a range of problematic behaviors. These behaviors ranged in their severity, their “fixability,” and whether they were inherent or acquired. These issues were not limited to issues in the clinical areas, but also included non-clinical duties. There was variability in whether residents with “fixable” problems should be considered problem residents. There was universal agreement that residents who caused disruption or turbulence within a program were problem residents. This turbulence extended to both the clinical and non-clinical settings.

The ABIM definition of “a trainee who demonstrates a significant enough problem that requires intervention by someone of authority, usually the program director or chief resident” does not capture the nuance reflected in this data. This definition centers around the need for intervention and does not encompass the severity of the issue, its effect on those around the resident, and the response to intervention. In our data, there were residents that many PDs considered to be residents with problems, not problem residents, who would be labeled as problem residents using the ABIM definition.

An ideal definition should acknowledge the diversity of thresholds used by PDs as well as incorporate the importance of disruption. Conceptualizations of “problem residents” in EM should consider those residents with a negative sphere of influence beyond their personal struggle. That negative influence can be limited to the program leadership, who are struggling to remediate the resident, or extend to impact the entire department. This framework allows for variation between PDs’ educational philosophies and environments, as well as incorporation of the concept of turbulence.

In our study, PDs consistently expressed an unwillingness to terminate residents even when there was persistent concern about their ability to practice independently. This reluctance to terminate learners is consistent with other studies in both undergraduate and graduate medical education,^13^,^14^ where progress, promotion, or graduation are rarely made on attributes other than grades.^15^ This “failure to fail”^14^,^16^ results in potentially unqualified physicians being allowed to practice and shifts the risks to the future patients and the responsibility to the state medical boards.

In the face of the combination of unfixable problems and an unwillingness to terminate learners, several PDs in our study advocated for a “front-door solution.” This approach focuses on the prevention of the selection of applicants with unfixable problems, thereby preventing them from entering the front door. Although this approach may seem like common sense, it is not the current practice.^17^ The majority of PDs focus on academic performance and do not emphasize professionalism during the application screening, even when the information is available.^18^–^21^ PDs instead rely heavily on the residency interview to identify personality issues,^22^ despite its lack of sensitivity for detecting problem applicants.^23^

Even for those who would like to implement this approach, there are major barriers to identifying these issues at the “front door.” The residency application can highlight exceptional performance in humanism, professionalism, and interpersonal communication, but issues or concerns are rarely expressed.^24^,^25^ When medical schools report concerns in the Medical Student Performance Evaluations (MSPE) they employ “linguistic gamesmanship” in an effort to “obfuscate rather than to inform the reader.”^19^ PDs are also overwhelmed with data about academic performance in the Electronic Residency Application Service (ERAS*®*) with little data about “professionalism, integrity, teamwork, and reliability.”^18^ PDs also have concerns about the difficulty and time needed for application review as well as the potential decrease in the academic potential of the residency.^26^

In our data, the unfixable problems were often related to issues surrounding professionalism and the lack of inter-personal skills. Unfortunately, it continues to be a challenge to identify applicants who will go on to have issues in these domains. Currently many groups are working to improve the quantity and quality of the data about medical student professionalism and inter-personal skills. Most notably, the Association of American Medical Colleges (AAMC) developed the Standardized Video Interview (SVI) as a tool to identify both high- and low-performing applicant proficiency in the core competencies of professionalism and inter-personal communication.^27^ Additionally, the AAMC MSPE Task Force called for the MSPE to do the following:

“Standardize, to the extent possible, information in the MSPE across schools, and present it clearly, concisely, and in a way that allows it to be easily located Include details on professionalism—both deficient and exemplary performance.”

Similarly, the Council of Residency Directors Standard Letter of Evaluation (SLOE) Task Force has modified the SLOE to provide greater information about issues such as work ethic, teamwork, and communication. Although there is a clear momentum toward improving the quantity and quality of this data, it is unclear if PDs value and trust, or are willing to act on this information. It is unclear if these changes will also lead to the improved identification of applicants who will go on to be problem residents.

## LIMITATIONS

Our study cohort consisted of a convenience sample of PDs. As a result, there was an over-representation of four-year, urban academic EM programs. Due to the nature of the semi-structured interviews, the interviews focused on details surrounding one or two memorable examples, leading to a recall bias that may have skewed the data toward the most extreme or most recent. However, we believe that this allowed us to gather detailed accounts, which provided sufficient specificity to adequately describe “problem residents.”

## CONCLUSION

These findings are a step toward classifying problem residents in EM. While they had different thresholds for what constituted a “problem,” PDs defined a problem resident differently than existing definitions. They characterized issues of clinical performance as either fixable or unfixable, and inherent or acquired. PDs particularly struggled to resolve behaviors that caused turbulence within a residency or department. We hope that our study adds nuance to the overall discussion across specialties. Additionally, we hope that the description of the fixable and unfixable problems will give all of the stakeholders the confidence to collectively create “front-door solutions” to the benefit of the resident, the medical community, and society.^14^
